# *Caenorhabditis elegans* as a Biological Model for Multilevel Biomarker Analysis in Environmental Toxicology and Risk Assessment

**DOI:** 10.5487/TR.2008.24.4.235

**Published:** 2008-12-01

**Authors:** Jinhee Choi

**Affiliations:** Faculty of Environmental Engineering, College of Urban Science, University of Seoul, Seoul, 130-743 Korea

**Keywords:** *Caenorhabditis elegans*, Multilevel biomarker, Environmental toxicology

## Abstract

While in some instances, loss of diversity results from acute toxicity (e.g. major pollution incidents), in most cases it results from long-term sub-lethal effects that alter the relative competitive ability and fitness of certain organisms. In such cases the sub-lethal effects will cause a physiological response in the organism that ultimately leads to community level changes. Very sensitive tools are now available to study sub-lethal responses at the molecular level. However, relating such laboratory measurements to ecological effects represents a substantial challenge that can only be met by investigation at all scales (molecular, individual organism and community level) with an appropriate group of organisms. Among the various in vertebrates which can be used as model organisms in such a way, the soil nematode, *Caenorhabditis elegans* appear to be a promising biological model to diagnose environmental quality. This paper reviews the current status of multilevel biomarkers in environmental toxicology, and *C. elegans* as promising organisms for this approach.

## INTRODUCTION

The reduction in point source pollution and the ban of some persistent chemicals have had positive effects on the level of environmental pollution over the last few decades. However, non-point source pollution by organic (e.g. pesticides, dioxins) and inorganic (e.g. heavy metals) compounds is still a global matter of concern. Moreover, numerous new industrial compounds have been synthesized for commercial and industrial purposes, which have generated environmental concerns, due to their high production and widespread use. Despite of the dramatic increase in the use of these chemicals, little information is available on their potential toxic effects on human and environmental health. The potential harmful effects on human and environmental health should be identified for the safe use of these chemicals. However, pollutions induced by these chemicals are caused by a complex mixture of compounds, making the exhaustive analyses of the contaminants present in polluted environments impossible, which limit the possibility of intensive toxicological studies (Risso-de-Faverney *et al.*, 2001). Therefore, rapid and sensitive tools are needed for screening hazardous properties of such chemicals prior to intensive toxicological investigation and risk assessment. Short-term bioassay systems would appear to be relevant for the preliminary screening of the potential effects of environmental chemicals on human and environmental health. Identification of suitable biological model is therefore, required, for the development of effective toxicity screening system. Various factors need to be considered for selecting a model system for this purpose, including knowledge of its biochemistry, physiology and of its demoecology, the availability of laboratory rearing protocols, etc. The soil nematode, *Caenorhabditis elegans* fulfills those criteria.

*C. elegans* is a ubiquitously distributed free-living nematode that lives mainly in the liquid phase of soils. It is the first multicellular organism to have its genome completely sequenced. The genome showed an unexpectedly high level of conservation with the vertebrate genome, which makes *C. elegans* an ideal system for biological studies, such as those in genetics, molecular biology, neurobiology, and development biology (Brenner, 1974; Bettinger *et al.*, 2004; Leacock and Reinke, 2006; Schafer, 2006; Schroeder, 2006; Antoshechkin and Sternberg, 2007). These same features have led to an increasing use of *C. elegans* in toxicology, as well as, in environmental toxicology (Leung *et al.*, 2008). In this review, multilevel biomarkers and the use of *C. elegans* as a model for this approach will be discussed in the context of environmental toxicology and risk assessment. *C. elegans* as a screening model system for prediction of mammalian toxicity will also be discussed.

***Mutlilevel biomarker in environmental toxicology and risk assessment.*** In environmental toxicology and ecotoxicology, substantial efforts have been devoted to developing and applying biomarkers for early warning indicators that respond before measurable effects on individuals and populations occur and also for identifying the causes of observed population- and community-level effects. Advances in molecular biology are extending the biomarker at the gene level (i.e., ecotoxicogenomics), whereas older biomarkers focused on measures of organism physiology or biochemistry. However, the extent to which biomarkers are able to provide unambiguous and ecologically relevant indicators of exposure to or effects of toxicants remains highly controversial (Forbes *et al.*, 2006). Although biomarkers can be helpful for gaining insight regarding the mechanisms causing observed effects of chemicals on whole-organism performance and may, in some cases, provide useful indicators of exposure, individual biomarker responses can not provide useful predictions of relevant ecological effects. Suites of biomarkers are only likely to provide increased predictability if they can be used in a comprehensive mechanistic model that integrates them into a measure of fitness (Forbes *et al.*, 2006). Recently, gene expression as an environmental stress response has been increasingly used in ecotoxicology, as it offers high sensitivity and mechanistic values to diagnose environmental contamination (Snell *et al.*, 2003; Lee *et al.*, 2006; Roh *et al.*, 2006, 2007; Poynton *et al.*, 2007). Genes up- or down-regulated in response to acute stress may predict chronic effects on individuals and populations before any such effect is apparent. Thus components and sometimes pathways that underlie physiological processes can be identified and investigated and aid further understanding of the mode of action of stressors. Stressor-specific signatures in gene expression profiles could offer a diagnostic approach to identify the cause of pollution event (Heckmann *et al.*, 2008). However, relating such laboratory measurements to ecological effects represents a substantial challenge that can only be met by investigation of response at all scales (molecular, individual organism and community level) with an appropriate group of organisms. Pollutant-induced molecular-, biochemical effects may potentially have consequences at higher levels of biological organization, such as changes in population dynamics or in biological diversity at both the intra- and interspecific levels and such changes may have adverse ecological consequences (Caquet *et al.*, 2000). Therefore, multilevel biomarker approach, evaluating different biological responses ranging from molecular to population/community level, would be more conservative for useful environmental monitoring (Lagadic *et al.*, 1994; Russo and Lagadic, 2000; Choi *et al.*, 2002; Lee and Choi, 2006; Lee *et al.*, 2008).

The multilevel biomarker concept is originally based on the fact that biological responses of an organism in natural environment progresses through homeostasis, compensatory and repair phases, as the exposure level or duration increases (Depledge, 1994). While an organism is exposed to contaminants, physiological compensatory mechanisms become active and changes in physiological processes or functions occur, which indicate that exposure has occurred. If the exposure persists or the level of exposure increases, these compensatory mechanisms become overwhelmed, damages occur, and physiological repair mechanisms become active. Under natural environmental conditions, as an organism progresses through these phases, the energy allocated for natural maintenance is reduced as more energy is needed for compensatory response and repair. The organism weakens and may be quickly eliminated from the population. Therefore, *in situ* survey of populations may not allow to detect diseased organisms even though exposure and effects have occurred (Newman and Jagoe, 1996). In the context of the multiple-response paradigm, the objective is not to quantitatively measure the amounts of different toxicants, but to determine where an organism is located on the continuum between homeostasis and disease. Responses indicate whether the organism is challenged but readily coping with toxicant stress (compensatory phase) or is deeply stressed and needs to use its energy resources to repair damages. This approach is essential to determine the general health status of the organism; moreover, it makes possible to extrapolate the relationship between responses at different levels of biological organization (Fossi *et al.*, 2000).

***C. elegans as a model for environmental toxicology.*** *C. elegans* is a good animal model for developing multilevel biomarker and multiscale analysis in ecotoxicology. Due to its abundance in soil ecosystems, its convenient handling in the laboratory, and its sensitivity to different kinds of stresses, *C. elegans* is frequently used in ecotoxicological studies utilizing vari-

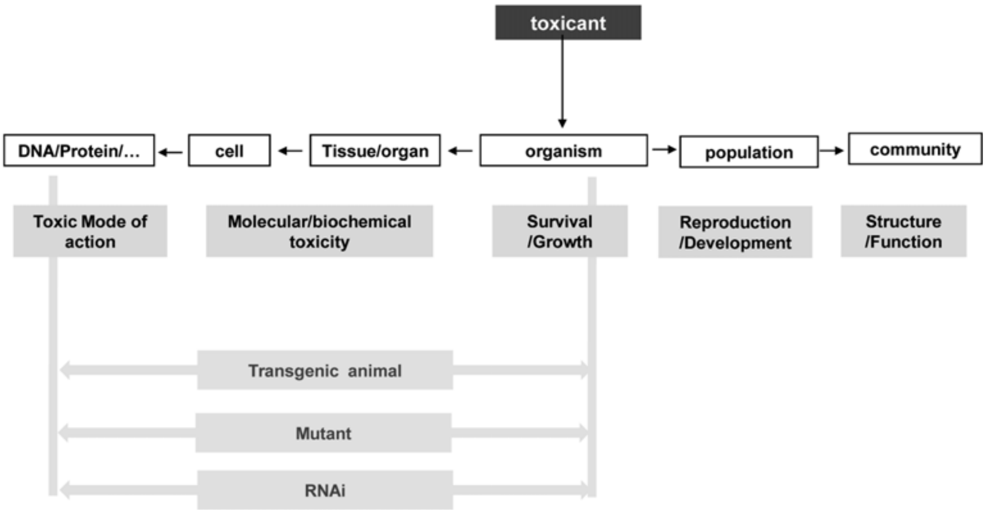

**Fig. 1.** Potential use of *C. elegans* as a model for environmental toxicology.

ous exposure media, including soil and water (Peredney and Williams, 2000; Williams *et al*., 2000; Boyd and Williams, 2003; Roh *et al*., 2006, 2007; Roh and Choi, 2008). As an *in vivo* model, *C. elegans* enables the detection of endpoints from molecular throughout organism/population levels (Fig. 1). *C. elegans* research area for multiscale analysis covers from molecular level to field-based ecotoxicology. The use of the responses of stress-related gene expression, functional genomics, transgenic biosensor has considerable potential for sensitive diagnosis of environmental contamination, and that *C. elegans* seems to be a good biological model for this approach: 1) Development of molecular tools includes study of nematode genomics and metabolomics in relation to environmental change, development of suitable biomarkers for environmental risk assessment, and development of nematode biosensors, etc. 2) Laboratory toxicity study using *C. elegans* covers estimation and optimisation of sub-lethal toxicity end points for risk assessment. 3) Field based nematode ecotoxicology area is to understand how nematode communities respond to environmental change in ecosystems, and how these

**Table 1.** Multiscale research area using *C. elegans* and the nematode

| Molecular tools for biomarkers | Laboratory toxicity assays | Field based ecotoxicology |
|---|---|---|
| - Testing of biosensor nematode strains in samples from appropriate field sites in terrestrial, freshwater and marine habitats.<br>- Investigation on the genotoxic effects of toxicants.<br>- Correlation of toxicity assays with genetic fingerprint profiles of populations.<br>- Usage of DNA arrays for gene expression analysis in response to environmental changes.<br>- Standardized robust molecular protocols for environmental quality analyses.<br>- Development of suitable biomarkers & biosensors for monitoring environmental changes | - Evaluation of the predictive value of established toxicity assays in environmental risk assessment.<br>- Comparison of variation of biological responses in laboratory established microcosms.<br>- Optimisation of whole sediment toxicity testing.<br>- Standardisation of laboratory assays with nematodes for contaminated soils/sediments and pure chemicals spiked into reference soils.<br>- Whole sediment exposure to pollutants to compare whole organism and molecular methods.<br>- Measurement of nematode fitness under varying short term pollution or other stresses.<br>- Validation of transgenic.<br>- *C. elegans* using field samples.<br>- Optimised risk assessment toxicity assays. | - Nematode community analyses as tools in risk assessment of environment changes.<br>- Determination of the relationship between complexity of nematode communities and food web complexity, functioning and stability.<br>- Linking of the field test to laboratory studies to measure communities.<br>- Standardisation of protocols and methods in nematode community analyses.<br>- Improved and optimised tools for environmental risk assessment.<br>- Development of quantitative indices for nematode community analyses.<br>- Improvement of understanding of ecosystem function. |

responses can in turn indicate changes in ecosystem function (Table 1). Even though, *C. elegans* is a suitable model for multiscale analysis from molecular to organism/population level, the range of *C. elegans* studies in environmental toxicology have been focused mostly on organism-level endpoints, such as mortality,

**Table 2.** Summary of chemical toxicity tests performed on *C. elegans*

| Chemicals | Toxic endpoints | References |
|---|---|---|
| $CdCl_2$ | lethality | Williams and Dusenbery, 1990; Dhawan *et al*., 2000; Barsyte *et al*., 2001; Ura *et al*., 2002; Chu *et al*., 2005; Ibiam and Grant, 2005; Roh *et al*., 2006. |
| | behavior, feeding behavior | Dhawan *et al*., 2000; Anderson *et al*., 2001; Ibiam and Grant, 2005. |
| | growth | Anderson *et al*., 2001; Ibiam and Grant, 2005; Stürzenbaum, 2007; Harada *et al*., 2007; Dong *et al*., 2008. |
| | reproduction development | Anderson *et al*., 2001; Swain *et al*., 2004; Ibiam and Grant, 2005; Hughes and Stürzenbaum, 2007; Harada *et al*., 2007; Dong *et al*., 2008. |
| | life span | Swain *et al*., 2004; Harada *et al*., 2007; Hughes and Stürzenbaum, 2007. |
| | sequence/ functional analysis | Dong *et al*., 2008. |
| | RNA/DNA ratio | Ibiam and Grant, 2005. |
| | gene expression | Barsyte *et al*., 2001; Liao *et al*., 2002; Swain *et al*., 2004; Roh *et al*., 2006; Cui *et al*., 2007. |
| | microarray/GO/KEGG | Cui *et al*., 2007. |
| $Pb(NO_3)/PbCl_2$ | lethality | Williams and Dusenbery, 1990; Dhawan *et al*., 2000; Ibiam and Grant, 2005; Roh *et al*., 2006. |
| | behavior, feeding behavior | Dhawan *et al*., 2000; Anderson *et al*., 2001. |
| | movement, growth, reproduction | Anderson *et al*., 2001; Ibiam and Grant, 2005. |
| | gene expression | Roh *et al*., 2006. |
| | RNA/DNA ratio | Ibiam and Grant, 2005. |
| $K_2Cr_2O_7$ | lethality | Williams and Dusenbery, 1990; Roh *et al*., 2006. |
| | gene expression | Roh *et al*., 2006. |
| $NaAsO_2$ | lethality | Williams and Dusenbery, 1990; Roh *et al*., 2006. |
| | stress-related gene expression | Roh *et al*., 2006. |
| $CuCl_2/CuSO_4$ | lethality | Williams and Dusenbery, 1990; Dhawan *et al*., 2000; Barsyte *et al*., 2001; Ibiam and Grant, 2005. |
| | behavior/feeding behavior | Dhawan *et al*., 2000; Anderson *et al*., 2001. |
| | growth, reproduction, lifespan | Anderson *et al*., 2001; Ibiam and Grant, 2005; Harada *et al*., 2007. |
| | RNA/DNA ratio | Ibiam and Grant, 2005. |
| $Al(NO_3)_3$ | lethality, behavior | Williams and Dusenbery, 1990; Dhawan *et al*., 2000 |
| $ZnCl_2/ZnSO_4$ | lethality | Williams and Dusenbery, 1990; Dhawan *et al*., 2000; Ibiam and Grant, 2005. |
| | behavior | Dhawan *et al*., 2000. |
| | RNA/DNA ratio | Ibiam and Grant, 2005. |
| $BaCl_2$ | body size, life span, DAF-16::GFP hsp-16.2, SOD, catalase activity | Wang *et al*., 2008. |
| DEHP | lethality, growth, reproduction microarray, gene expression | Roh *et al*., 2007. |
| Chloropyrifos | lethality, growth, reproduction enzyme activity, gene expression | Roh and Choi, 2008. |
| Nano-Platinum | lifespan, oxidative stress resistance | Kim *et al*., 2008. |

behavior, growth, or reproduction. Most of chemical toxicity tests performed on *C. elegans* listed in Table 2.

***Gene expression.*** The application of DNA microarrays to toxicogenomics links toxicological effects of exposure with expression profiles of several thousand genes. The gene expression profiles are altered during toxicity, as either a direct or indirect result of toxicant exposure and the comparison of numerous specific expression profiles facilitates the differentiation between intoxication and true responses to environmental stressors. The application of microarrays provides the means to identify complex pathways and strategies that an exposed organism applies in response to environmental stressors. Gene expression profiles obtained by DNA microarrays are also believed to provide amore comprehensive, sensitive and characteristic insight into toxicity than typical toxicological parameters such as morphological changes, altered reproductive capacity or mortality. In addition to these classical (eco)toxicological parameters, (eco)toxicogenomics is a powerful tool that unravels mechanistic processes, reveals novel modes of action, and provides the opportunity to get a dynamic picture of biological systems and the ability to comprehensively dissect different states of biological activities in cells, tissues or whole organisms (Steinberg *et al.*, 2008). Due to the availability of the whole genome sequence, *C. elegans* has long been subject to gene expression studies. Microarrays using *C. elegans* have been conducted on steroid hormones (Custodia *et al.*, 2001), Polychlorinated biphenyls (PCBs; Menzel *et al.*, 2007), di(2-ethylhexyl)phthalate (DEHP; Roh *et al.*, 2007) and cadmium (Cui *et al.*, 2007). Among those chemicals, effect of cadmium has been most intensively investigated. The DNA microarray experiments on cadmium by Cui *et al.* (2007) identified 237 up-regulated and 53 downregulated genes that significantly changed following either 4 h or 24 h exposure to cadmium. These genes were clustered into early and late response genes. The former encompasses pathways, which regulate the localization and transportation of differen chemical species (in particular metal ions). Recently, the functional relations of gene expression and phenotypic response have been widely investigated (Dong *et al.*, 2005; Roh *et al.*, 2007; Roh and choi, 2008).

***Functional genomics.*** *C. elegans* is an attractive animal model for the study of the ecotoxicological relevance of chemical-induced gene-level responses (Menzel *et al.*, 2005; Reichert and Menzel, 2005). Functional genomic tools, such as, mutant and RNAi, can offer the possibility to assess the physiological meaning of up- or down-regulated gene expression by chemical exposure and can provide indicators of the toxic mode of action from the level of a single gene to that of the whole organism (Menzel *et al.*, 2007). The results of gene expression analysis can be validated *in vivo* using mutational approaches in *C. elegans*. (Kwon *et al.,* 2004; Menzel *et al.,* 2007). A rich collection of mutant makes *C. elegans* a particularly attractive animal model. Sensitive mutants can be used to improve the sensitivity of toxic response and thus have high potential for screening a toxicity of chemicals in a relatively short time (Chu *et al.*, 2005). Mutant *C. elegans* can be used to confirm the role of specific molecular targets based on gene expression analysis (Menzel *et al.*, 2007).

***Biosensor.*** Transgenic *C. elegans* biosensor has been developed to monitor environmental stress. The use of transgenic animals is not a new approach in environmental toxicology. Fish transgenic model has been developed and received much attention and its promising capability was demonstrated (Jones *et al.*, 1996; Kurauchi *et al.*, 2005; Scholz *et al.*, 2005; Stringham and Candido, 2005). Nonetheless, most of the protocols require skills-based, long, and costly experiments, which make them difficult to adapt for the rapid routine assessment of field samples. *C. elegans* allows the preparation of a large number of staged and genetically homogeneous animals in the laboratory in a short time. The advantage of a rich collection of gene engineering approaches and well-established transgenesis approaches also presents a short cut to the development of a sensitive biosensor that other organism models cannot surpass. Indeed, different promoters (e.g., hsp and mt) and alternative reporters (e.g., GFP, beta-galactosidase, and luciferase) have been tested in different transgenic designs (Roesijadi 1994; Yoshimi *et al.*, 2002; Chu *et al.*, 2005). Bioavailability and toxicity of a wide range of pollutants have been investigated using transgenic *C. elegans* biosensors (Power and de Pomerai, 1999; Lagido *et al.*, 2001; Dengg and van Meel, 2004; Roh *et al.,* 2006, 2007). Sensitivity of *C. elegans* to many heavy metals is similar to that of mammals (Williams and Dusenbery, 1988) indicating potential for evaluating toxicity to humans. *C. elegans* biosensors represent a more complex level of biological organisation and a higher trophic level than the bacterial and yeast luminescent biosensors already available (Paton *et al.*, 1997; Hollis *et al.*, 2000). This is pertinent when predicting toxicity to humans or implications for environmental health, as this approach can be used more generally to evaluate *C. elegans* metabolic status (Lagido *et al.*, 2001).

***C. elegans* as a screening model for prediction of mammalian toxicity.** Recently, the growing awareness of the possibility of using wildlife animals as sentinels for human environmentally-induced diseases has created a demand for biomarkers that are nonlethal and correlate with adverse effects in humans (Kendall *et al.*, 2001). Links between wildlife and human health can serve as a premise for extrapolation in risk assessment. Indeed, humans share many cellular and subcellular mechanisms with wildlife species. Humans and wildlife also overlap in their environments and may therefore be exposed to the same contaminants. There is evidence to suggest that when highly conserved systems are targeted by environmental toxicants, both ecosystem and human health suffer (Kendall *et al.*, 2001). Biomonitoring organisms have long been used as a means of warning people of unsafe environments. There is increasing evidence that this is the case both at the level of genetic and physiological similarity, and at the level of actual toxicity data. The role of *C. elegans* is particularly valuable in this regard. *C. elegans* is considered an ideal system for understanding mammalian pathology, including toxicity. Because, many of the basic physiological processes and stress responses that are observed in higher organisms are conserved in *C. elegans*. Moreover, the genome of *C. elegans* shows an unexpectedly high level of conservation with the vertebrate genome (Brenner, 1974; Bettinger *et al.*, 2004; Leacock *et al.*, 2006; Schafer, 2006; Schroeder, 2006). Therefore, by conducting molecular analyses of the response of conserved pathways to *in vivo* chemical exposure, toxicity data obtained in *C. elegans* may provide an insight into the mammalian toxicity.

Conserved genome and signaling pathways are particularly interesting as an alternative model for prediction of mammalian toxicity. *C .elegans* homologues have been identified for 60~80% of human genes (Kaletta and Hengartner 2006). Many signal transduction pathways are conserved in nematodes and vertebrates (i.e. Wnt pathway via β-catenin; Receptor serine/threonine kinase pathway; Receptor tyrosine kinase; Notch-Delta pathway; Receptor-linked cytoplasmic tyrosine kinase pathway; Apoptosis pathway; Receptor protein tyrosine phosphatase pathway; G-protein coupled receptor pathway; Integrin pathway Cadherin pathway; Gap junction pathway; Ligand-gated cation channel pathway) (NRC, 2000; Leung *et al.*, 2008). Pathways relevant to oxidative stress, such as, the p38 MAPK and AKT signaling cascades, the ubiquitin-proteasome pathway, and the oxidative stress response pathway are also conserved in the worm (Leiers *et al.*, 2003; Grad and Lemire, 2004; Ayyadevara *et al.*, 2005; Inoue *et al.*, 2005; Kipreos, 2005; Gami *et al.*, 2006; Daitoku and Fukamizu, 2007; Wang *et al.*, 2007; Ayyadevara *et al.*, 2008; Tullet *et al.*, 2008). Additionally, the main neurotransmitter systems (cholinergic, GABAergic, glutamatergic, dopaminergic, and serotoninergic) and their genetic networks (from neurotransmitter metabolism to vesicle cycling and synaptic transmission) are phylogenetically conserved from nematodes to vertebrates, which allows for findings from *C. elegans* to be extrapolated and further confirmed in vertebrate systems (Leung *et al.*, 2008).

Moreover, genome-wide screening, which can serve as a hypothesis-finding tool, providing a direction for further mechanistic investigation, is possible in *C. elegans* using forward genetics, DNA microarrays, or genome-wide RNAi. This approach is particularly useful for studying any toxicant with a poorly understood mechanism of action. Forward genetics screen, a useful method in mechanistic toxicology, is efficient in *C. elegans* because the mutants can cover genes expressed in a variety of tissues. A genome-wide RNAi screen, typically assesses a number of physiological parameters at the same time thereby facilitating the interpretation of screening results, are also being used for discovering gene function (Leung *et al.*, 2008).

The use of *C. elegans* as a predictive model for human toxicity was studied by estimating LC50 values of heavy metals exposure (Williams and Dusenbery, 1988), and by investigating the behavioral toxicity of

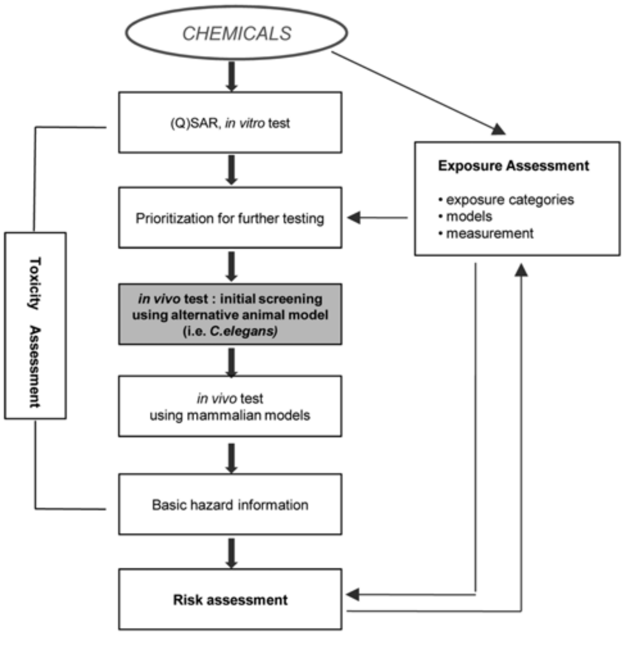

**Fig. 2.** Potential use of *C. elegans* as an alternative animal model for initial screening of chemical toxicity.

organophosphorous pesticides (Anderson *et al*., 2004; Cole *et al*., 2004). Several other studies have also validated a number of *C. elegans*-based assays for predicting neurological and developmental toxicity in mammalian species (Khanna *et al*., 1997; Dhawan *et al*., 1999; Williams *et al*., 2000; Anderson *et al*., 2004). Comparative toxicity study with *C. elegans* has been most exploited to date, using neurologically active chemicals (Leung *et al*., 2008). Overall results form comparative toxicity studies suggest that *C. elegans* may react to chemicals with enough similarity to mammals to be useful as a first-round screening agent for toxicity (Fig. 2).

***Concluding remarks.*** To better diagnose environmental quality, multilevel biomarkers-based approach, which permits better understanding of the impact of pollutants on organisms, should be implemented in environmental monitoring procedures. Moreover, the interconnections between ecologic heath and human health should not be overlooked. What is needed, in the future, are new and innovative approaches that integrate effects across different levels of biological complexity and provide a clear understanding of all the hazards posed by environmental pollution, not only to ecological systems but for human health as well. *C. elegans* seems to be a powerful model for this approach. Especially, as complement system to *in vitro* and *in vivo* vertebrate models, *C. elegans* seems to have a high potential to be a good candidate for an alternative animal model for mammalian toxicity screening study.
